# Plumbagin Alleviates Intracerebroventricular-Quinolinic Acid Induced Depression-like Behavior and Memory Deficits in Wistar Rats

**DOI:** 10.3390/molecules27061834

**Published:** 2022-03-11

**Authors:** Mandeep Kumar Arora, Anish Ratra, Syed Mohammed Basheeruddin Asdaq, Ali A. Alshamrani, Abdulkhaliq J. Alsalman, Mehnaz Kamal, Ritu Tomar, Jagannath Sahoo, Jangra Ashok, Mohd Imran

**Affiliations:** 1 Department of Pharmacology, KIET School of Pharmacy, Ghaziabad 201206, India; mandeepk.arora@dituniversity.edu.in (M.K.A.); anisharatra2@gmail.com (A.R.); ashok@cuh.ac.in (J.A.); 2 School of Pharmaceutical and Population Health Informatics, DIT University, Dehradun 248171, India; ritu.tomar@dituniversity.edu.in (R.T.); director.pharmacy@dituniversity.edu.in (J.S.); 3 Department of Pharmacy Practice, College of Pharmacy, AlMaarefa University, Diriyah, Riyadh 13713, Saudi Arabia; 4 Department of Pharmacology & Toxicology, College of Pharmacy, King Saud University, Riyadh 11451, Saudi Arabia; aaalshamrani@ksu.edu.sa; 5 Department of Clinical Pharmacy, Faculty of Pharmacy, Northern Border University, Rafha 91911, Saudi Arabia; kaliqs@gmail.com; 6 Department of Pharmaceutical Chemistry, College of Pharmacy, Prince Sattam Bin Abdulaziz University, Al-Kharj 11942, Saudi Arabia; mailtomehnaz@gmail.com; 7 Department of Pharmaceutical Sciences, Central University of Haryana, Mahendergarh 123031, India; 8 Department of Pharmaceutical Chemistry, Faculty of Pharmacy, Northern Border University, Rafha 91911, Saudi Arabia; imran.pchem@gmail.com

**Keywords:** quinolinic acid, hippocampus, plumbagin, oxidative–nitrosative stress

## Abstract

Plumbagin, a hydroxy-1,4-naphthoquinone, confers neuroprotection via antioxidant and anti-inflammatory properties. The present study aimed to assess the effect of plumbagin on behavioral and memory deficits induced by intrahippocampal administration of Quinolinic acid (QA) in male Wistar rats and reveal the associated mechanisms. QA (300 nM/4 μL in Normal saline) was administered i.c.v. in the hippocampus. QA administration caused depression-like behavior (forced swim test and tail suspension tests), anxiety-like behavior (open field test and elevated plus maze), and elevated anhedonia behavior (sucrose preference test). Furthermore, oxidative–nitrosative stress (increased nitrite content and lipid peroxidation with reduction of GSH), inflammation (increased IL-1β), cholinergic dysfunction, and mitochondrial complex (I, II, and IV) dysfunction were observed in the hippocampus region of QA-treated rats as compared to normal controls. Plumbagin (10 and 20 mg/kg; p.o.) treatment for 21 days significantly ameliorated behavioral and memory deficits in QA-administered rats. Moreover, plumbagin treatment restored the GSH level and reduced the MDA and nitrite level in the hippocampus. Furthermore, QA-induced cholinergic dysfunction and mitochondrial impairment were found to be ameliorated by plumbagin treatment. In conclusion, our results suggested that plumbagin offers a neuroprotective potential that could serve as a promising pharmacological approach to mitigate neurobehavioral changes associated with neurodegeneration.

## 1. Introduction

Free radical generation leading to nitro-oxidative stress and neuroinflammation are considered as the major cause of neurobehavioral changes and depression-like behavior [1]. Neurobehavioral changes are caused primarily by any brain injury that will have a severe impact on a patient’s day-to-day functioning and social involvement, leading to depression [2]. An earlier report suggests that, globally, depression is the fourth leading source of disability [3]. In India, women and the elderly are at a high risk of depression, mostly among widows [4].

Quinolinic acid (QA) is a neurotoxic biosynthetic product of tryptophan, obtained in nano concentrations through the kynurenine pathway [5]. QA is known to be an endogenic agonist of the N-Methyl-D-aspartate (NMDA) receptor, which is involved in memory function. Elevated concentrations of QA in the brain were found to be involved in the pathogenesis of various neuronal diseases including depression, cognitive deficits, Huntington’s disease, and Alzheimer’s disease [6,7,8]. The administration of QA through intrastriatal injection can induce Huntington disease-like symptoms in rodents [9]. On the other hand, intraventricular infusion of QA can produce learning and memory deficits in rats [10]. Administration of QA directly into the brain, enhances Ca^2+^ influx on binding to NMDA receptors and causes excitotoxic damage to the hippocampus and striatal regions [11]. In addition to its excitotoxic action through the NMDA receptor, it also directly enhances the release and decreases glutamate uptake, an excitotoxic neurotransmitter [5]. QA augments free radical generation and leads to oxidative–nitrosative stress associated with neuro-inflammation [12,13]. Apart from this, QA was also found to elicit cell apoptosis through OH-induced DNA damage and lipid peroxidation by forming a complex with iron [14]. In addition, QA alters mitochondrial function by inducing oxidative damage to mitochondrial DNA and lipids, leading to cell death followed by neurobehavioral changes [14]. Intracerebroventricular (ICV) injection of QA is associated with neurobehavioral changes [15].

Plumbagin, a naphthoquinone derivative extracted from the roots of medicinal plant plumbago zeylanica [16], was reported to have neuroprotective potential along with other therapeutic properties including anti-bacterial, antidiarrheal, and wound properties [17,18,19,20]. Mounting evidence suggests that plumbagin also possesses anti-oxidative properties by blocking free radical generation as well as anti-inflammatory potential by decreasing the levels of inflammatory mediators [21,22]. Findings by Pinho et al. demonstrated that plumbagin prevented NO production [23]. Taken together, these studies indicate that plumbagin significantly decreases oxidative damage and inflammation in neuronal cells, thus ameliorating neurobehavioral changes resulting from DNA damage. Thus, findings from the aforementioned experimental studies warrant the determination of the pharmacological efficacy of plumbagin against QA-induced neurobehavioral anomalies. We consider that ICV administration of QA causes neurochemical changes in the hippocampus that lead to neurobehavioral changes. In addition, we discovered the potential neuroprotective effect of plumbagin against QA-induced depression-like behavior and memory deficits.

## 2. Results

### 2.1. Effect of Plumbagin on Immobility Time during the Forced Swim Test

**Figure 1:**
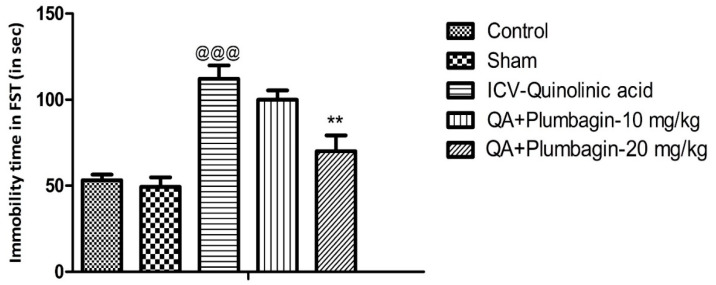
Effect of plumbagin on QA-induced alterations in immobility time during forced swim test. Data represent mean ± SEM (*n* = 6). ^@@@^ *p* < 0.001 as compared control group; ** *p* < 0.01 as compared to QA-induced group.

The Forced swim test was assessed in all the experimental groups. We observed that the ICV-QA-exposed group shows remarkable (*p* < 0.001) prolongation of immobility time in comparison with the normal control set. Treatment of QA-exposed group with plumbagin-10 mg/kg did not significantly change the immobility time as compared with the ICV-QA-exposed group. Interestingly, QA + plumbagin-20 mg/kg displayed a noteworthy (*p* < 0.01) decrease in the immobility time when compared with the ICV-QA-exposed group (Figure 1).

### 2.2. Effect of Plumbagin on Depression-like Behavior in the Tail Suspension Test

**Figure 2:**
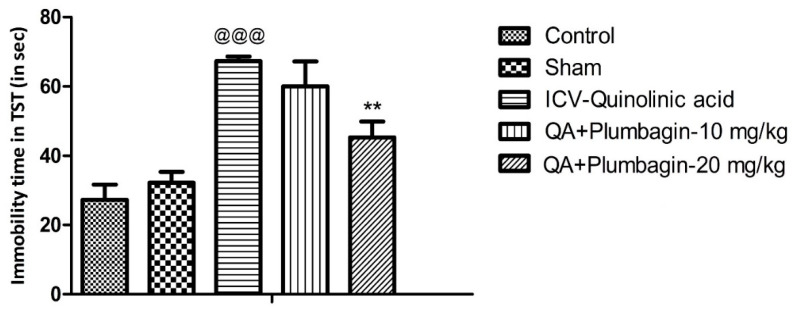
Effect of plumbagin on QA-induced increased in immobility time during the Tail Suspension Test. Data represent mean ± SEM (*n* = 6). ^@@@^ *p* < 0.001 as compared to control group; ** *p* < 0.01 as compared to QA-induced group.

To verify plumbagin’s ability to reduce depression-like behavior, we conducted the Tail Suspension Test as described in the methods section. The ICV-QA-treated group showed a significant increase (*p* < 0.001) in the immobility time compared to the controls. In agreement with the FST results (Figure 1), no significant impact was observed in animals treated with 10 mg/kg plumbagin. The 20 mg/kg dose on the other hand showed a remarkable (** *p* < 0.01) decrease in the immobility time when equated to the ICV-QA-exposed group (Figure 2).

### 2.3. Effect of Plumbagin on Sucrose Consumption Test

**Figure 3:**
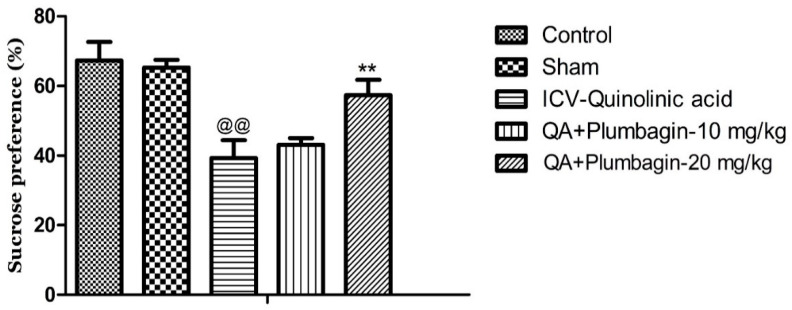
Effect of plumbagin on QA-provoked decline in percent of sucrose preference during the Sucrose Preference Test. Data represent mean ± SEM (*n* = 6). ^@@^ *p* < 0.01 as compared to control group; ** *p* < 0.01 as compared to QA-administered group.

To further examine the potential anti-depressive impacts of plumbagin, we assessed the rats’ interests toward palatable foods (e.g., sucrose) and the levels of consummatory pleasure (hedonic value) using the Sucrose Preference Test. We observed that the ICV-QA-exposed group displayed a marked reduction in their sucrose consumption (^@@^ *p* < 0.01) compared to the normal control group. Treatment with 10 mg/kg dose of plumbagin had no significant impact on the level of consumption when compared with the ICV-QA-exposed group. Plumbagin 20 mg/kg significantly (** *p* < 0.01) increased the percent of sucrose preference in contrast to the ICV-QA treated-group (Figure 3). These results suggest that plumbagin reverses loss of reward–sensitivity and thus depression-like behavior resulted from QA exposure.

### 2.4. Effect of Plumbagin on Elevated Plus Maze

**Figure 4:**
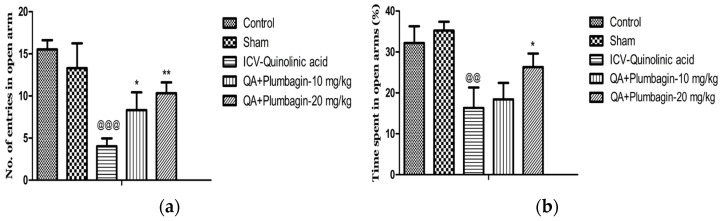
(**a**) Effect of plumbagin on QA-induced decrease no. of entries in open arms in EPM; (**b**) Effect of plumbagin on QA-induced diminution in percent time consumed in exposed arms during EPM. Data represent mean ± SEM (*n* = 6). ^@@^ *p* < 0.01 as compared to control group; ^@@@^ *p* < 0.01 as compared to control group; * *p* < 0.05 and ** *p* < 0.01 as compared to QA-induced group.

To assess social isolation and thus anxiety-type behaviors, we performed the EPM test. In this test, anxiety is measured by the number of times or the proportion of time these rats remain in the closed arms of the maze, without meeting the open areas. Figure 4a,b show that in comparison to the normal control group, in the ICV-QA-treated group we observed a significantly (*p* < 0.001) decreased number of entrances and time spent (*p* < 0.01) in exposed arms. The low dose of plumbagin 10 mg/kg shows a significant (*p* < 0.05) elevation in the number of passes with no effect on the time spent in the exposed arms in comparison with ICV-QA-treated group. Additionally, a high dose of plumbagin 20 mg/kg remarkably (*p* < 0.01) results in an increase in the number of entrances and the time spent (*p* < 0.05) in the open arm compared to ICV-QA-exposed group.

### 2.5. Effect of Plumbagin on Open Field Test

**Figure 5:**
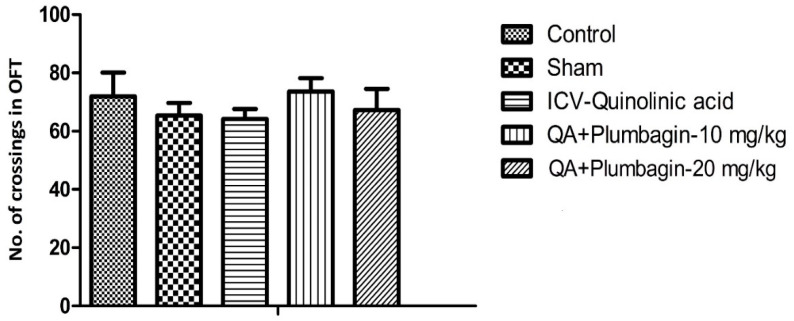
Effect of plumbagin on QA-induced effect on number of crossings in OFT through the Open Field Test. Data represent no significant effect when compared to control and the ICV QA-induced group.

In the OFT, we found that number of crossings were not altered in different experimental groups. Plumbagin treatment (10 and 20 mg/kg) showed non-significant alteration in comparison to the ICV-QA-exposed group (Figure 5).

### 2.6. Effect of Plumbagin on ICV QA-Induced Differences in Reduced Glutathione (GSH) Level

**Figure 6:**
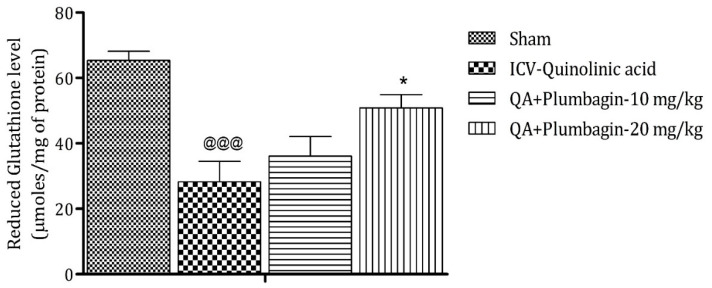
Effect of plumbagin on QA-induced variations in level of GSH for the determination of antioxidant activity in the rat hippocampus. Data represent mean ± SEM (*n* = 6). ^@@@^ *p* < 0.001 as compared to sham group; * *p* < 0.05 as compared to QA-induced group.

Analysis after sacrificing the animal model shows that reduction in GSH level is a marker of neuronal damage in the brain. The ICV-QA-exposed group shows a noteworthy (*p* < 0.001) decrease in the concentration of GSH as contrasted with the sham group. A low dose of plumbagin of 10 mg/kg shows no significant effects in comparison with the ICV-QA-exposed group. In contrast, a high dose of plumbagin of 20 mg/kg remarkably (* *p* < 0.05) increases the level of GSH when contrasted with the ICV-QA-exposed group (Figure 6).

### 2.7. Effect of Plumbagin on ICV QA-Induced Differences in AChE Level

**Figure 7:**
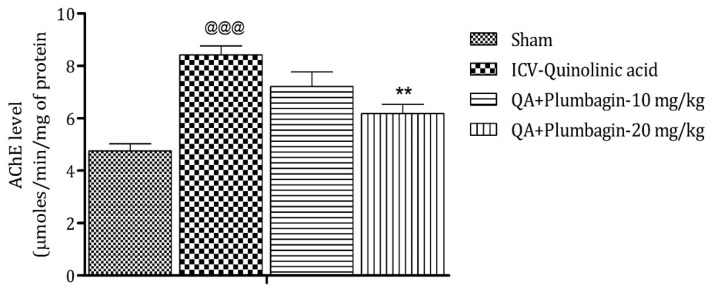
Effect of plumbagin on QA-induced variations in level of AChE for the determination of memory functions in rats. Data represent mean ± SEM (*n* = 6). ^@@@^ *p* < 0.001 as compared to sham group; ** *p* < 0.01 as compared to QA-induced group.

Assessment of AChE depicts that its elevated level is a marker of memory deficits, and its decreased level maintains memory functions [24]. In comparison to the sham group, the ICV QA-exposed group markedly (*p* < 0.001) increases the level of AChE. After treatment with a low dose of plumbagin of 10 mg/kg, a non-significant effect was observed; whereas with treatment with a high dose of plumbagin, the level of AChE decreased significantly (*p* < 0.01) on comparison with the ICV QA-induced group (Figure 7).

### 2.8. Effect of Plumbagin on ICV QA-Induced Differences in Nitric Oxide (NO) Level

**Figure 8:**
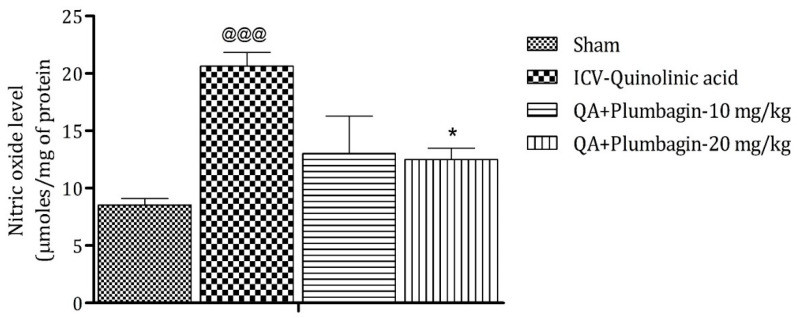
Effect of Plumbagin on QA-induced variations in level of NO in rats. Data represent mean ± SEM (*n* = 6). ^@@@^ *p* < 0.001 as compared to sham group; * *p* < 0.05 as compared to QA-induced group.

Elevated level of NO leads to the generation of free radicals followed by neurodegeneration. The ICV QA-induced group showed a significant (^@@@^ *p* < 0.001) increase in the concentration of NO in comparison with the sham group. No substantial effect was observed by the low dose of plumbagin of 10 mg/kg. However, a high dose of plumbagin remarkably (*p* < 0.05) lowered the NO concentration when contrasted with the ICV QA-exposed group (Figure 8).

### 2.9. Effect of Plumbagin on ICV QA-Induced Differences in Malondialdehyde (MDA) Level

**Figure 9:**
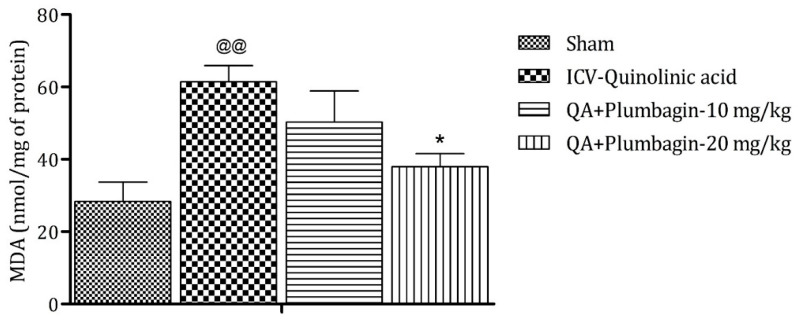
Effect of plumbagin on QA-induced variations in level of MDA in rats. Data represent mean ± SEM (*n* = 6). ^@@^ *p* < 0.01 as compared to sham group; * *p* < 0.05 as compared to QA-induced group.

On analysis it was found that MDA is a marker of oxidative stress. In the ICV QA-treated group the concentration of MDA was markedly (*p* < 0.01) increased in comparison with sham group. A plumbagin low dose of 10 mg/kg shows non-significant effects in the level of MDA and a plumbagin high dose of 20 mg/kg depicts a noteworthy (*p* < 0.05) decrease in the concentration of MDA when contrasted with the ICV QA-treated group (Figure 9).

### 2.10. Effect of Plumbagin on ICV QA-Induced Differences in Mitochondrial Complexes I, II, and IV

**Figure 10:**
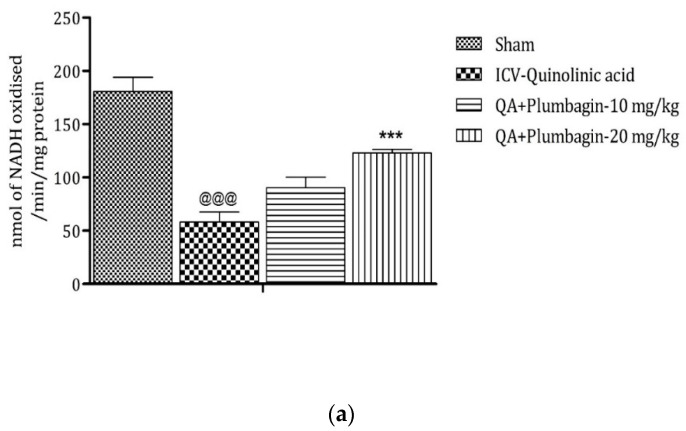

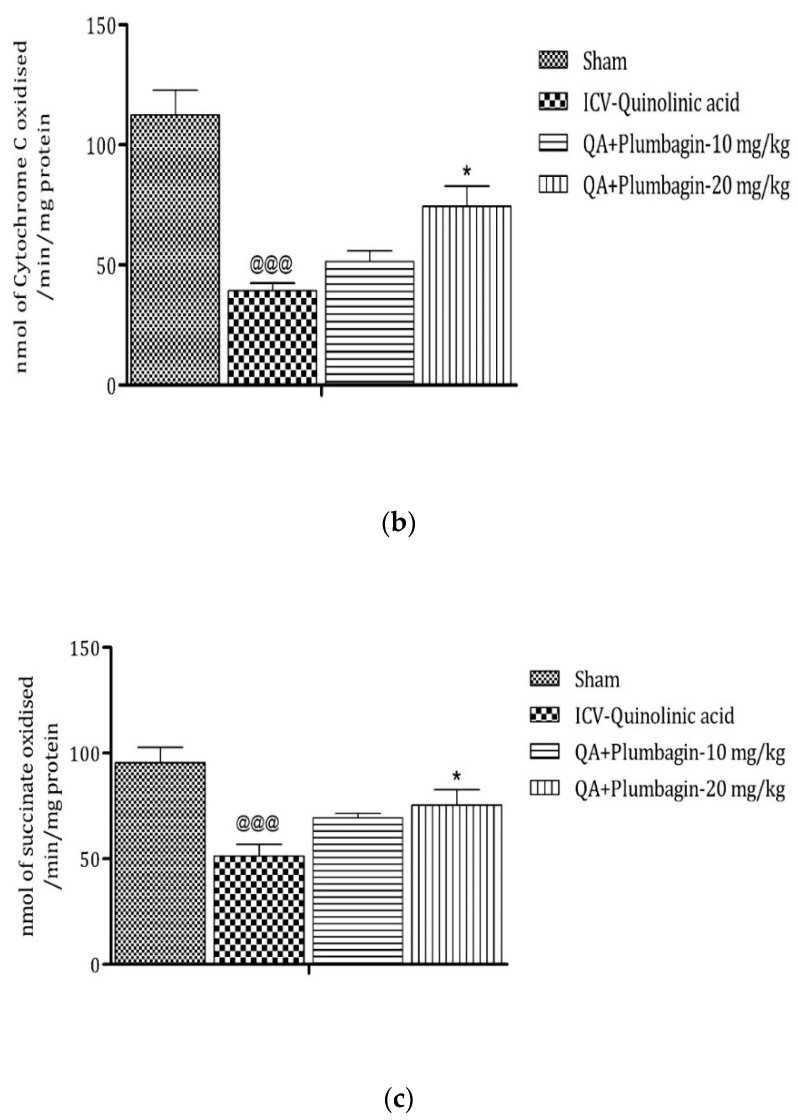
(**a**) Outcome of plumbagin on QA-induced variations in level of Complex I in rats. Data represent mean ± SEM (*n* = 6). ^@@@^ *p* < 0.001 as compared to sham group; *** *p* < 0.001 as compared to QA-induced group. (**b**) Outcome of plumbagin on QA-induced variations in level of Complex II in rats. Data represent mean ± SEM (*n* = 6). ^@@@^ *p* < 0.001 as compared to sham group; * *p* < 0.05 as compared to QA-induced group. (**c**) Outcome of plumbagin on QA-induced variations in level of Complex IV in rats. Data represent mean ± SEM (*n* = 6). ^@@@^ *p* < 0.001 as compared to sham group; * *p* < 0.05 as compared to QA-induced group.

Mitochondrial complexes are involved in energy generation in the electron transport chain cycle. The ICV QA-exposed group displays a noteworthy (*p* < 0.001) reduction in levels of complex I, II, and IV respectively in comparison with the sham group. Low dose plumbagin 10 mg/kg shows non-significant effects in the concentration of these complexes when compared to the ICV QA-exposed group. Treatment with high dose plumbagin 20 mg/kg increases the concentration of complex I (*p* < 0.001) as compared to the ICV QA-exposed group. A high dose of plumbagin of 20 mg/kg shows a remarkable (*p* < 0.05) increase in the concentration of complexes II and IV, respectively, in comparison with the ICV QA-exposed group, Figure 10a–c.

### 2.11. Effect of Plumbagin on ICV QA-Induced Differences in IL-1β Level

**Figure 11:**
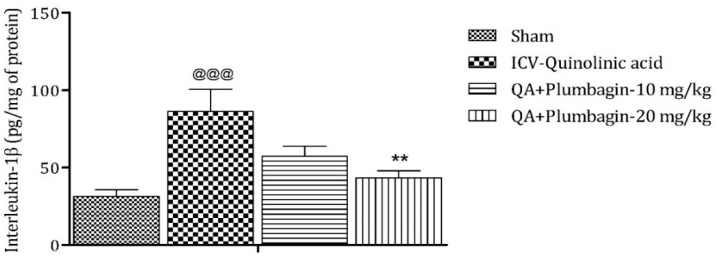
Effect of plumbagin on QA-induced variations in level of IL-1β in rats. Data represent mean ± SEM (*n* = 6). ^@@@^ *p* < 0.001 as compared to sham group; ** *p* < 0.01 as compared to QA-induced group.

IL-1β is an important mediator of inflammatory response and is involved in numerous pathophysiological conditions. Both animal and human experimental studies suggested that IL-1β is a key mediator in the pathogenesis of depression [25]. A previous study demonstrated that knock-down of the IL-1β gene by lentivirus can inhibit LPS-induced anxiety and depressive-like behavior [26]. In the current investigation, we found an elevated level of IL-1β (*p* < 0.001) in the ICV QA-treated group when compared with the sham group. Low dose plumbagin 10 mg/kg shows non-significant effects and high dose of plumbagin 20 mg/kg reveals marked (*p* < 0.01) diminution in IL-1β concentration in relation with the ICV QA-exposed group (Figure 11).

## 3. Discussion

The purpose of the present research is to explore the fundamental mechanism of plumbagin and its neuronal defensive potential on QA-induced neuronal behavior changes. The outcome of the research suggests that plumbagin remarkably ameliorates the QA-induced neurobehavioral changes and memory deficits in Wistar rats. Neurodegenerative disorders are the predominant root of behavioral variations [27]. From prior research it was elucidated that neurobehavioral changes are related to oxidative–nitrosative stress [1,28]. In addition to this, our recent study also found that oxidative–nitrosative stress is accompanied with behavioral changes. The resulted neuronal behavioral variations are markedly ameliorated by the oral administration of plumbagin, demonstrating its potential of assuaging oxidative stress, lipid peroxidation, anxiety behavior, weakened mitochondrial functioning, anhedonia behavior, and memory discrepancies. The anti-oxidative potential of plumbagin as well as its ability of elevating the level of antioxidants along with decreasing the generation of free radicals are the motive of its neuroprotective effects.

QA is a potent neurotoxin and has been shown to have a significant effect on neuronal behavior [29,30,31]. QA is well known to be an agonist of the NMDA receptor which on stimulation exaggerates the concentration of calcium leading to excitotoxic cell death resulting in numerous neurobehavioral changes [32,33]. An experimental model taking into account the ICV administration of QA is well-known for causing behavioral alterations by provoking oxidative stress, neuroinflammation, lipid peroxidation, and, ultimately, cell apoptosis. Earlier experimental studies revealed the potential of QA for being involved in prompting oxidative–nitrosative stress accompanied by the malfunctioning of mitochondria [34]. Various behavioral parameters are evaluated for assessing the neuroprotective potential of plumbagin against QA-induced neuronal changes. The Forced Swim Test and Tail Suspension Test exemplify escape-aimed behavior and are utilized to evaluate the depression-like behavior. In addition to this sucrose preference test is utilized for estimating the anhedonia performance. The results of elevated plus maze along with open filed test manifest the anxiety inducing behavior of QA. All in together discloses that the intrahippocampal administration of QA grades in depression-like neuronal behavior which was demonstrated by behavioral parameters on comparing normal and sham control groups. Consequently, our conclusions are in accordance with previous studies signifying that intrahippocampal injection of QA results in behavioral variations [5,35,36]. In addition to this our results also indicate that plumbagin administration significantly improves the neurobehavioral alterations together with anxiety-like behavior, neuronal inflammation, and memory deficiencies induced by QA administration reflecting enhanced functioning of the hippocampus. In fact, in our previous study, we have reported the neuroprotective and acetylcholinesterase inhibitory activity of plumbagin in ICV-LPS induced neurobehaviorally deficient rats [37]. In addition, the neuroprotective potential of plumbagin is in agreement with other prior studies [38,39].

Numerous studies demonstrate the association between the oxidative stress, misfunctioning of mitochondria and neurobehavioral changes [40,41]. Imbalance between antioxidants and oxidants provokes the generation of free radicals, which was found to be the major cause of oxidative stress and lipid peroxidation. ICV QA injection induces oxidative–nitrosative stress via generation of free radicals. In accordance with this our present study concludes that QA is a chief source of prompting oxidative–nitrosative stress in hippocampus. Earlier studies also revealed that the ICV injection of QA is responsible for inducing oxidative–nitrosative stress in the hippocampus [36,42]. QA is also involved in the induction of lipid peroxidation which establishes a complex with iron which results in OH radical generation [43]. In addition to this, our outcomes demonstrate that QA provokes an elevated level of MDA, a marker of lipid peroxidation and along with this the level of NO was also found to be amplified in the hippocampus after the administration of QA. From past studies it was reported that plumbagin, due to its potential of oxidizing (reduced nicotinamide adenine dinucleotide phosphate) NADPH, results in the prevention of lipid peroxidation due to the non-availability of NADPH for the assistance of peroxidation [44]. In addition to this, various other studies revealed that plumbagin have the potential of elevating GSH levels [45]. Our present study also showed that plumbagin increases the level of GSH, an endogenous antioxidant, which demonstrates its anti-oxidative effect; therefore, our study suggested that plumbagin has the potential of ameliorating oxidative–nitrosative stress induced by intrahippocampal administration of QA and these outcomes are concomitant with the above-mentioned studies.

Mitochondria, an organelle responsible for generating energy, is also considered a target of initiating oxidative stress. QA having an excitotoxic potential undergoes an elevated level of calcium which in turns causes misfunctioning of mitochondria after binding the NMDA receptor. Numerous studies proposed that the interaction of QA with mitochondria results in transmutation of its DNA, enhanced membrane porousness, and oxidative–nitrosative stress [33,34]. Moreover, the misfunctioning of mitochondrial complexes I, II, and IV can also be accompanied by energy deficiency via mitochondria. Severely improper functioning of complexes II and IV are chiefly implicated in the generation of free radicals followed by oxidative mitochondrial injury [34]. In accordance with this our results demonstrate that the intrahippocampal injection of QA results in the decreased level of mitochondrial complexes I, II, and IV. Our extant study shows that plumbagin remarkably elevates the level of mitochondrial complexes I, II, and IV by ameliorating oxidative–nitrosative stress thereby showing its potential of improving the functions of mitochondria in the hippocampus.

## 4. Materials and Methods

### 4.1. Test Animals

Male Wistar Rats (200–250 g) were purchased from the National Institute of Biologicals (NIB), Noida, India, for the experiment. The research protocol (IAEC/KSOP/E/19/01) was approved by the institutional animal ethics committee (IAEC) of KIET, India. Animals were housed at optimal temperatures (23 ± 2 °C) with 50–70% relative humidity on an illuminated and unilluminated cycle for 720 min with adequate nutritional content, according to conventional laboratory settings. Prior to the experiment, the animals were acclimated for one week. Wet animal beddings posed a constant threat of contamination, so beddings were regularly replaced.

### 4.2. Experimental Grouping and Dosing

Fifty Wistar rats (male) were randomly allotted to five different experimental groups (Table 1), namely, Control (Normal Saline), sham control, QA group (300 nM/4 μL in Normal saline), low dose plumbagin group (10 mg/kg p.o.) with QA, and high dose plumbagin group (20 mg/kg p.o.) with QA. The dose of QA and plumbagin was selected on the basis of previous experimental studies [46,47,48].

### 4.3. Study Design

**Figure 12:**
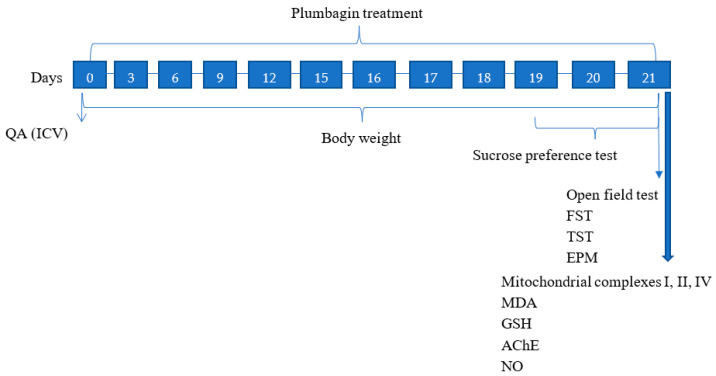
Schematic illustration of the experimental study timeline.

The experimental study lasted for 21 days in which QA was administered on Day 0, and plumbagin treatment was given at different time periods throughout the study as indicated in Figure 12. The behavioral parameters were assessed on the last three days, and biochemical parameters were evaluated upon study completion in the hippocampus of the rats following cervical dislocation.

### 4.4. Drugs and Chemicals

Plumbagin and QA (pyridine-2,3-dicarboxylic acid) were purchased from Sigma-Aldrich Corp., Saint Louis, MO, USA. Other required synthetic compounds (sodium lauryl sulphate, trisodium citrate, thiobarbituric acid, DTNB, potassium dihydrogen orthophosphate, sodium hydrogen phosphate, EDTA, disodium hydrogen phosphate, and sodium chloride) were obtained locally. Plumbagin was suspended in 20% Tween-80.

### 4.5. Surgical Procedure (Intracerebroventricular Injection)

A solution of 70% solution was used for the sterilization of all the surgical equipment to prevent any infection due to surgery. Before the initiation of the experimental procedure, Wistar rats (200–250 g) were anesthetized by using ketamine (70 mg/kg *i.p*) to avoid any suffering during the entire procedure, and body temperatures were maintained to prevent hypothermia. When the rat became unconscious, it was placed in the stereotaxic apparatus where its head was positioned with the help of the ear bars. After ensuring no movements of the rat’s head, the hairs were shaved entirely between the area of the shoulder and eyes, followed by disinfecting the shaved area by using 70% ethanol and iodine. By using a surgical incisor, a small incision was made over the shaved area and a hole was drilled in the skull. To administer QA, a sterilized Hamilton’s syringe containing 10 µL QA was used. To prevent the flow of QA outside the hole, the syringe remained placed in that position for around 5 min. The incision was then sutured followed by the application of Betadine^®^ ointment along with Neosporin^®^ powder. The rat was routinely observed for a period of 1 week after the surgery, and, at the end of 8–10 days, the sutures were removed to avoid any discomfort [49].

### 4.6. Behavioral Parameters

#### 4.6.1. Forced Swim Test (FST)

FST is the gold standard utilized for the evaluation of depressive-like conditions. A cylindrical tank (30 cm height and 20 cm diameter) was used for performing the experiment. The tank was filled with water up to 15 cm of height from the bottom of the tank. The water temperature was maintained at 23 °C by using a thermometer. After achieving the optimal temperature, the rat was held gently from the tail and placed inside the water-filled tank. The activity of rats was observed for 6 min, after that in the last 5 min the total immobility time (in sec) was measured. Immobility time reveals the escape-oriented behavior [50].

#### 4.6.2. Tail Suspension Test (TST)

A TST was performed to assess depression-like behavior as previously demonstrated [51]. Briefly, adhesive tape (Around 17 cm) was used, and individual rats were hanged from a height of 40 cm. The tape was applied at the tail end in such a manner so that 2–3 cm of the tail remained untapped. Each rat was suspended for a period of 6 min and during the last 5 min immobility time (in sec) was measured [50].

#### 4.6.3. Sucrose Preference Test

The Sucrose Preference Test was used for the assessment of anhedonia behavior. In this test, two bottles, one filled with normal drinking water and another with sucrose solution, were used. Before the initiation of the experiment, the rats were administered with sucrose solution (2%) and normal water to check desire for sucrose. Subsequently, the rats were freely allowed to drink either of the bottles for 8 h. The positions of the bottles were altered from time to time to avoid any biasing. Observations were made by addressing the percentage of sucrose preference [52].

#### 4.6.4. Elevated Plus Maze (EPM)

The EPM test was used for assessing anxiety-like behavior. Elevated plus maze apparatus (50 cm above the floor) consists of two open (35 × 5 cm^2^) and two closed (35 × 5 cm^2^) arms perpendicular to each other along with a minor center square (5 × 5 cm^2^). During the experiment, rats were individually placed at the middle of the maze, with the head directed towards the open arm. Rats were allowed to get familiarized for around 5 min. Finally, at the end of the experiment, the overall number of entries and amount of time spent in open arms was recorded [1].

#### 4.6.5. Open Field Test (OFT)

An OFT was conducted to assess behavioral changes. In this test an acrylic transparent open field test compartment (72 × 72 × 36 cm^3^) is partitioned into 16 quadrants (18 × 18 cm^2^) of similar dimensions. The central four compartments and 12 side compartments are denoted as the center and the periphery, respectively. Rats were individually positioned in this OFT apparatus. Crossings in the center and periphery, and the rearing activities were recorded for a period of 10 min with the help of a video camera [53].

### 4.7. Biochemical Parameters

#### 4.7.1. Preparation of Tissue Homogenate

Tissue homogenates were prepared on the 21st day of the experimental protocol after sacrificing the rats of each group. The hippocampus was isolated on an ice-cold petri dish. A concentration of 0.1 M PBS (pH 7.4) was used to form 10% homogenate. The resulting homogenates were centrifuged for 15 min and the supernatants were collected and kept at −20 °C for further analysis.

#### 4.7.2. Lipid Peroxidation Estimation

Lipid peroxidation in the hippocampus was estimated after sacrificing the Wistar rats on the 21st day of the experimental study. Hippocampus homogenate (50 μL) was mixed meticulously with 50 μL of SDS (8.1%). In the resultant mixture (350 μL) acetic acid (20%) and thiobarbituric acid (0.8%) were added, and water was incorporated to sustain the volume at 1.5 mL. The solution was heated at 95 °C for 1 h, then cooled at room temperature and centrifuged at 10,000× *g* for 10 min to gather the supernatant. The absorbance was recorded at λmax 532 nm [54].

#### 4.7.3. Reduced Glutathione (GSH) Estimation

GSH was estimated using the Ellman’s method [55]. Briefly, the supernatant and 10% TCA (ratio 1:1) were mixed and centrifuged for 10 min at 4 °C. Isolated supernatant liquid was collected in which 0.3 M Na_2_HPO_4_ (1000 μL) and Ellman’s reagent (250 μL) was added. Finally, absorbance was measured at λmax 412 nm.

#### 4.7.4. AChE Estimation

Assessment of memory deficiency was achieved by measuring AChE [56]. This method required a preparation of a mixture acetyl choline iodide (1 µM), 5,5-dithio-bis-(2-nitrobenzoic acid) (2 µM), PBS (100 µM) (pH 7), and homogenate of the hippocampus tissue sample. The resulted mixture was incubated for 10 min at 37 °C, then 500 μL of serine hemisulphate (0.5 µM) was added to break the reaction, and finally absorbance was measured at λmax 412 nm.

#### 4.7.5. Nitric Oxide Estimation

Griess reagent was mixed with hippocampal supernatant to estimate nitric oxide and was kept in an unilluminated place for 10 min at RT. Absorbance was measured (540 nm) for the resulted solution and results were reported as µmol/mg of protein [57].

#### 4.7.6. Isolation and Preparation of Mitochondria

The hippocampus was dissected out from the brain and mitochondria were isolated following previously reported protocols [58]. The isolated mitochondria were homogenized in the medium comprising sucrose (75 µM), 4-(2-hydroxyethyl)-1-piperazineethanesulfonic acid (HEPES) (5 µM), bovine serum albumin (1 mg/mL), mannitol (225 µM), and 1 µM ethylene glycol tetra acetic acid (1 µM). The resulted homogenized mixture was centrifuged at 2000× *g* for 3 min (4 °C). Pellets were collected after disposing of the supernatant and again resuspended and centrifuged. Subsequently, the synaptosomal layer was resuspended in digitonin (0.02%) and then centrifuged at 12,000× *g* for 10 min. Resulted mitochondrial pellets were used for downstream experiments.

#### 4.7.7. Complex I (NADH: Coenzyme Q Oxidoreductase)

Complex I is involved in the catalytic oxidation of NADH into NAD^+^ by reducing cytochrome C. A 3 mL solution of glycylglycine (0.2 µM), NADH (6 µM), sodium bicarbonate (0.02 µM) solution, and 1 µM of cytochrome C were mixed, and pH was maintained at 8.5. The sample was added and absorbance was calculated (550 nm) for 3 min. The activity of Complex I was evaluated, and the results are given in the form of nm of NADH oxidized per min per mg of protein [59].

#### 4.7.8. Complex II (SDH Activity)

For the estimation of Complex II activity, 0.6 M Succinic acid, phosphate buffer (0.2 M), 1% BSA, and potassium ferricyanide (0.03 M) of pH 7.8 were mixed. In the resultant mixture the sample was added, and absorbance was measured (420 nm) for 3 min. Finally, results are shown in the form of nm of ACH dehydrogenase per min per mg of protein [60].

#### 4.7.9. Complex IV (Assay of Cytochrome c Oxidase)

Complex IV activity was estimated by mixing reduced cytochrome C and Phosphate buffer. Afterwards, the sample was added, and absorbance was measured (550 nm) for 3 min. Results are demonstrated in the form of nm of cytochrome c oxidized per min per mg of protein [61].

#### 4.7.10. Interleukin-1β (IL-1β) Level Estimation

IL-1β was estimated by using ELISA Kit following the manufacturer’s protocol (Elabscience, India). Freshly prepared tissue homogenate was centrifuged at 14,000× *g* for 20 min at 4 °C, and the resulting supernatant was used for assessment. Absorbance was then measured at 450 nm and results are demonstrated as pg per mg of proteins. Total protein was quantified by following the procedure of the Lowry method. Bovine serum albumin was used as a standard in protein quantification [62].

### 4.8. Statistical Analysis

Statistical analysis was carried out with the help of GraphPad Prism software. Results are presented as mean ± standard error of mean (SEM). Means were compared by one-way ANOVA followed by the Tukey post hoc test. Results were considered significant at * *p* < 0.05 relative to the control.

## 5. Conclusions

In conclusion, the findings of this ongoing study demonstrate the neuroprotective effects of plumbagin against ICV QA-induced depression-like behavior and memory deficits. Plumbagin exhibits its neuroprotective potential by mitigating the oxidative–nitrosative stress by decreasing the levels of oxidative markers, namely, GSH, AChE, and NO and by raising the levels of mitochondrial complexes I, II, and IV, which are essential for energy generation. Plumbagin could be a novel therapeutic approach for governing neurobehavioral changes and memory deficits; an elaborative study, however, is needed to illuminate the detailed mechanism of plumbagin.

## Figures and Tables

**Table 1:** Experimental grouping and dosing.

Group I	Control (Normal Saline) (*n* = 10)
Group II	Sham control (*n* = 10)
Group III	QA (300 nM/4 μL QA (QA) in Normal saline) (*n* = 10)
Group IV	Plumbagin (10 mg/kg p.o. Low dose) + QA (300 nM/ 4 µL QA in Normal saline) (*n* = 10)
Group V	Plumbagin (20 mg/kg p.o. High dose) + QA (300 nM/ 4 µL QA in Normal saline) (*n* = 10)

## Data Availability

All data included in paper, no additional data available.

## Sample Availability

Samples of the compounds are not available from the authors.
